# Centering the Strengths of American Indian Culture, Families and Communities to Overcome Type 2 Diabetes

**DOI:** 10.3389/fpubh.2021.788285

**Published:** 2022-03-16

**Authors:** Melissa Walls, Rachel Chambers, Marissa Begay, Kristin Masten, Kevalin Aulandez, Jennifer Richards, Miigis Gonzalez, Angie Forsberg, Leonela Nelson, Francene Larzelere, Cindy McDougall, Megan Lhotka, Ryan Grass, Sidnee Kellar, Raymond Reid, Allison Barlow

**Affiliations:** Bloomberg School of Public Health, Johns Hopkins University, Baltimore, MD, United States

**Keywords:** CBPR, American Indian/Alaska Native, Native American, intervention–behavioral, diabetes, prevention

## Abstract

Type 2 diabetes (T2D) is a critical Indigenous health inequity rooted in experiences of colonization and marginalization including disproportionate exposure to stressors, disruption of traditional family and food systems, and attacks on cultural practices that have led to more sedentary lifestyles. Thus, an important step in redressing inequities is building awareness of and interventions attuned to unique Indigenous contexts influencing T2D and Indigenous culture as a pathway to community wellbeing. Using a dynamic, stage-based model of intervention development and evaluation, we detail the creation and evolution of a family-based, culturally centered T2D preventive intervention: Together on Diabetes (later Together Overcoming Diabetes) (TOD). The TOD program was built by and for Indigenous communities via community-based participatory research and has been implemented across diverse cultural contexts. The TOD curriculum approaches health through a holistic lens of spiritual, mental, physical and emotional wellness. Preliminary evidence suggests TOD is effective in reducing diabetes risk factors including lowering BMI and depressive symptoms, and the program is viewed favorably by participants and community members. We discuss lessons learned regarding collaborative intervention development and adaptation across Indigenous cultures, as well as future directions for TOD.

## Introduction

Type 2 diabetes (T2D) represents a critical health inequity in many American Indian (AI) communities where rates are more than double the overall US population (1), including in the Midwest and Southwest regions where our research primarily takes place (2–5). Diabetes is also a major player in cardiovascular disease, the leading cause of AI death (1), and contributes to complications that substantially reduce quality of life (6). Prevalence of T2D for AI youth is 7 times greater than White youth (1.2 vs. 0.17/1,000) (5) and 2.6 times more than US all races (1.2 vs. 0.46/1,000). Amidst such startling statistics are realities of human suffering reflected in excerpts from focus groups our team led with Ojibwe adults: “*I live with pain every day, if I don't take those pills every day my arm goes numb, gets useless.”* and “*I found out about 35 years ago that I had diabetes. I've lost a lot of my eyesight, I developed cataracts from the diabetes. I'm in the last stages of kidney disease.”* These stories motivate our goals of health promotion for and with current and future generations of AI people.

Doubly motivating are the roots of injustice underlying the T2D epidemic. Stress has been considered an etiological agent of T2D for centuries (7) and compromises disease management, healthy behaviors and metabolic control (8, 9). Many AIs experience heightened exposure to stressors including poverty, intergenerational trauma, and discrimination (10). A deep-rooted backdrop of these stressors and health inequities is historical trauma, the cumulative wounding from collective AI exposure to policies and practices perpetuated with ethnocidal intent (11–14). Notably, rates of AI T2D were low prior to the 1940s (15). Disruption of Indigenous family and sustenance systems via attacks on cultural practices, traditional languages, and relocation policies led to sedentary lifestyles and reliance on government commodity food programs (14) contributing to disparate T2D rates today. In sum, uniquely profound issues of social justice underpin this modern epidemic for AIs.

There is thus a critical need to address the fundamental causes (16, 17) of and risk/protective factors linked to T2D and its complications within AI communities. Behavioral interventions have been shown efficacious for improving outcomes related to chronic disease and result in treatment-related cost savings (18–20). For instance, the Special Diabetes Program for Indians Diabetes Prevention Program (DPP) aimed at reducing fat intake and promoting healthier diets and physical activity through individual counseling has seen remarkable success in terms of reductions in kidney failure, diabetic eye diseases, and stabilization of obesity rates among AIs (21, 22).

Our community/university team has worked to create, enhance, and evaluate a multi-generational preventive intervention for AI families within diverse tribal communities called Together on Diabetes and later Together Overcoming Diabetes (TOD). Our approach emphasizes lessons learned in prior T2D prevention and treatment programs and expands the approach to include family-centered, home-based holistic health promotion, community-specific targets for health promotion and risk, and addressing common barriers to care. A major tenet of the development, piloting, and ongoing trials for TOD is that Indigenous community perspectives and co-creation of preventive interventions are necessary for program effectiveness, acceptability, and sustainability. Indeed, authentic community engagement through orientations such as community-based participatory research (CBPR) are essential for ethically addressing health priorities within any community; such approaches also align with the sovereign right of Tribal Nations to govern and oversee any research that takes place within its territories (23).

Another tenet of our approach is that Indigenous Peoples have long centered cultural ways and the reclamation of traditional values and teachings as critical components of health promotion (24, 25). Yet, positivist science frameworks have lagged behind Indigenous wisdom with minimal (albeit growing) inclusion of Indigenous-specific programs in what is considered “evidence-based” (26). Incorporating Indigenous culture in preventive interventions also represents a redressing of historical trauma and a pathway to healing for T2D specifically wherein the disease has been understood in diverse Native contexts as a disruption of balance, loss of cultural ways, attacks and loss of traditional language, and/or contamination of lifeways due to colonization (27–29). Incorporating contemporary Native cultural context, such as the existence of food deserts, was also another valuable aspect of our approach to T2D prevention. For example, the Navajo Nation only has 13 grocery stores in a land area equivalent to New England (30). We aimed to address these issues by centering community and cultural contexts in the *process* and *content* of TOD curriculum creation and evaluation.

## A Framework for Iterative, Community-Driven Public Health Intervention Development and Evaluation

**Figure 1:**
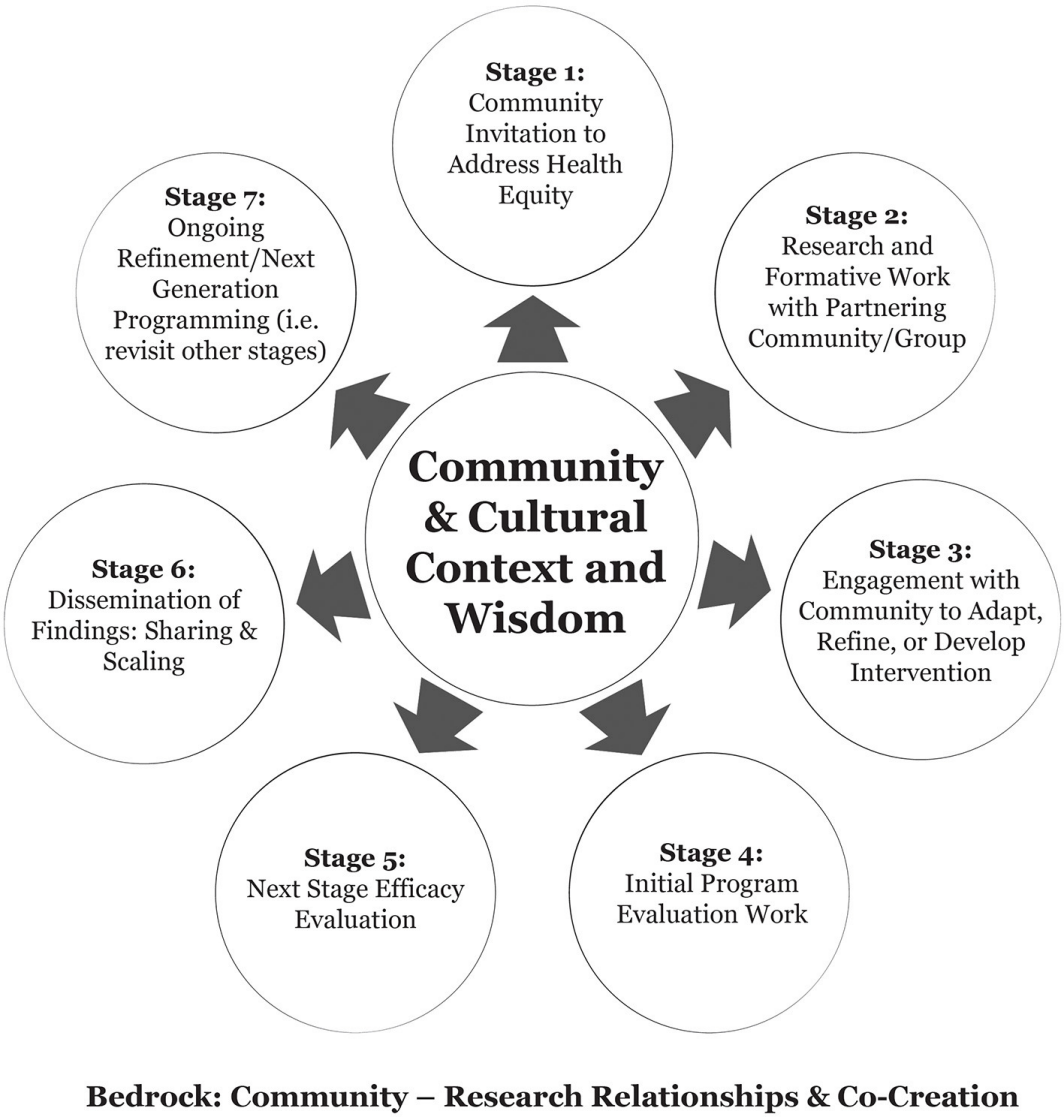
A stage-based model of tribally-based intervention development and evaluation.

As noted, our team's approach to public health intervention development and evaluation is rooted in community-researcher relationships and collaboration. Figure 1 displays a stage-based model of tribally-based intervention development and evaluation.

The framework includes considerations such as community identification of priorities, iterative, cyclical processes, and a range of intervention development and/or adaptation possibilities drawing from previous researchers working with Indigenous communities (31, 32). We consider and describe seven stages to our approach; importantly, stages may not neatly flow in numerical order, and some stages may not be clearly relevant to all iterations of intervention progression or community priorities. Keeping this in mind, and in alignment with CBPR, a common early stage (**Stage 1**) involves an invitation from a community to address an issue of concern. **Stage 2** prioritizes the identification of community and/or culturally relevant risk/protective factors related to the health outcome and/or formative work to identify issues relevant to the feasibility and/or acceptability of a proposed intervention. This is often accomplished through “basic” (vs. applied) or formative research techniques. Stage 3 considers a range of possibilities for intervention development or adaptation such as those outlined by Okamoto et al. (33); this could include building an intervention from the ground-up, implementing an existing program, and/or adapting a program to match local culture and context. Stages 4 & 5 represent a move to intervention implementation and evaluation; at Stage 4, initial evaluation work may be process oriented, collect pilot evaluation data, and/or focus more deeply on feasibility or acceptability. **Stage 5** represents “next phase” efficacy evaluation, which has a primary focus on understanding the impact of the program on intended outcomes. This may include mixed-methods, qualitative methods to identify nuanced mechanisms of program effectiveness, and/or quantitative methods including variations on western scientific experimental designs to isolate and evaluate the impacts of the intervention on outcomes. At **Stage 6**, partners focus on sharing results within the community where work has taken place, and later sharing and scaling lessons learned so other communities may benefit or use the program. **Stage 7** is an explicit nod to the fact that this process is dynamic; any “final” intervention may need to be agile to modification, re-evaluation, and so forth, and thus require a revisit to any or all previously completed stages. In the sections that follow, we detail our team's diverse pathways through these stages as part of two distinct iterations of the TOD program in the Southwestern and Great Lakes regions of the United States.

## Program Iteration #1: Tod_Southwest (SW)

### TOD_SW Stage 1: Invitation and Description of the Team

In 1980, a Johns Hopkins pediatrician, Dr. Mathuram Santosham, began work with the White Mountain Apache Tribe (WMAT) to address an outbreak of diarrheal disease in babies. Following this, he moved his family to the Fort Apache reservation where he lived and worked for over 5 years. Here, he worked with WMAT and later, the Navajo Nation to identify and address disparities in infectious disease among Native children. In 1991, he founded the Johns Hopkins University (JHU) Center for American Indian Health (CAIH) with a mission to *work in partnership with tribal communities to design public health programs that raise the health status, self-sufficiency, and health leadership of Native people to the highest possible level*. CAIH strives to use community-engaged approaches, especially CBPR, in all aspects of its work. Over the past 30+ years, JHUCAIH grown to also address behavioral and mental health inequities (e.g., suicide, early childhood health and well-being, obesity, etc.), including T2D.

In the late 2000s, Navajo and Apache communities identified T2D as an urgent issue and began to work with CAIH to identify strategies to address this concern. In Fall of 2011, CAIH researchers applied for and received funding to develop, implement and evaluate a program to address T2D with 1 Apache and 3 Navajo communities. The funding was flexible, providing the team the opportunity to adapt existing or develop new intervention content and structure, determine the target population for the intervention, and identify the most appropriate study design.

The TOD_SW team initially included an On-site Manager at the Albuquerque Hub of CAIH; local study teams comprised of 3–4 local Native staff at each of the 4 communities; Community Advisory Boards (CAB) from the 4 participating communities; a Cross Site Steering Committee (CSSC); and a Curriculum Specialist, Evaluator, Program Manager, and PI based in CAIH administrative offices in Baltimore, MD. We also consulted with a Harvard-based pediatrician with nutrition expertise, a diabetes expert from Indian Health Services (IHS) and various other experts across the partnering communities and the US. CABs at each site were created to guide program development and implementation. Each CAB consisted of 8–12 members and included health educators, teachers, law enforcement representatives, medical providers, behavioral health specialists, traditional practitioners/elders, parents, youth and community members from the study sites. The CSSC was comprised of pediatricians, nurse practitioners, diabetes consultants and educators, mental health providers, IHS clinic leaders, community-based program leads, diabetes focused program leads, cultural consultants, a traditional practitioner, elders, wellness center coordinators, and CAIH study staff from the four study sites. Many CSSC members also joined their local CABs. All study staff who worked on the TOD_SW study were hired as full-time employees of JHU. All community staff were Native; most were from the community in which they worked.

### TOD_SW Stage 2: Basic and Formative Research

Initial TOD_SW formative work took place over a period of 9 months, during which we aimed to build a culturally appropriate program that fit the unique needs of the four communities and adequately addressed existing gaps in T2D prevention and management. The CSSC met four times in the first 6 months of the project (2012) and annually thereafter; meetings were held in person, began with a prayer followed by lunch and a sharing of knowledge. Meetings ended with a closing prayer. At the first meeting included presentation from site representatives that include information about: (1) T2D prevalence and incidence data from their site/service unit, (2) T2D programming in their community, (3) perceived gaps and opportunities in T2D prevention, management and treatment, and (4) perceived diabetes risk and protective factors; the urgent need for programming for youth with diabetes or pre-diabetes emerged as a need across sites. Additional gaps included those at the service level (e.g., lack of standardized protocols for screening youth for T2D, need for culturally-centered care) and at the program/community level (lack of evidence-based materials for youth with T2D, need for family-centered programming, limited holistic training/holistic health focus for educators, inconsistent nutrition information about traditional foods, language/cultural disconnect barriers, predominant focus on negative vs. positive behaviors, lack of transportation services). Identified opportunities included enthusiasm of local partners, robust network of diabetes programming for adults, and traditional Native medicine programs at many of the participating sites.

CABs met quarterly throughout the duration of the study. CAB meetings incorporated the Indigenous holistic framework (34) as follows: *Respect*: CAB meetings ensured TOD was designed and implemented with respect for Navajo/Apache culture and communities. *Responsibility*: a wide range of community members were engaged consistently in order to develop and sustain meaningful relationships and foster a shared responsibility/commitment toward community wellness. Further, the impact of the TOD program was discussed at final CAB meetings. *Relevance*: CABs provided information about changing needs of the community and identified potential activities that could be implemented at community level. *Reciprocity*: CAB meetings engaged Native and non-Native community members and had a primary goal of shared learning. Resources exchanged at these meetings were also mutually beneficial. Community partners helped each other because we all had a shared goal of community wellness.

Early in the study the CSSC proposed roundtables with youth and parents in the four communities to better understand how to address T2D through team-identified gaps and opportunities. Roundtable guides were developed with CABs and Baltimore team members. The guides focused on healthy and unhealthy behaviors and knowledge and attitudes about physical, mental, spiritual and emotional health. Participating youth were also asked about their role models and who/what influences their behaviors. A total of 12 roundtables were completed with youth ages 10–19 years and nine with adult parents. Roundtables were facilitated by trained Navajo or Apache study staff who took notes which were reviewed and compiled to inform TOD. Roundtables were not recorded. Key takeaways were: (1) youth did not think holistically about the food they ate, (2) parents wanted to be involved in programming, as they had limited knowledge of healthy eating/exercise and wanted to support their children's lifestyle changes, (3) there were few activities available for families, and (4) youth needed additional support related to life skills (e.g., learning about problem solving and communication). A theme across roundtables was that parents play a key role in youth diets, physical activity, and mental health. Thus, TOD would be delivered to youth alongside their parents or trusted adults, named “Support Persons (SPs),” with a family/multi-generational, holistic health emphasis. A family focus also aligns with Navajo and Apache cultural norms, which are common across many Indigenous groups (35–37).

When TOD was being developed, few evidence-based diabetes prevention or management programs existed for Native populations (38). There were even fewer programs for youth with or at risk for T2D and none that met the criteria of being family-centered, home-based, and/or tailored by and for Native populations. The study team identified and reviewed four programs that met at least one of these specifications: (1) The *Diabetes Prevention Program (DPP*) which has a rich evidence base for reducing incidence of diabetes among at-risk adults (39). At the time, the DPP had been adapted into the adult-focused Native Lifestyle Balance Program–designed for Native adults. (2) The TODAY (Treatment Options for Diabetic Youth and Young Adults) study curriculum, designed for youth with T2D, was undergoing a rigorous efficacy trial at the time the TOD program was being developed (40). (3) *Cherokee Choices*, a school and community-based diabetes prevention program designed for Native youth and their parents (41), was undergoing a pilot feasibility trial. (4) CAIH programs that were home-based, for families, designed for Native populations and efficacious, such as the Family Spirit program (42). Ultimately, we determined that none of these programs fully met the gaps and built on the opportunities identified across our target communities. As such, we decided to use the DPP program as a model of TOD structure and utilize other programs to inspire curriculum content.

### TOD_SW Stage 3: Program Adaptation and Refinement

Information from the CSSCs, CABs, and roundtables was coupled with knowledge from local study staff and scientific experts in order for a Curriculum Specialist and Study Manager (Curriculum Team) to draft a basic outline of 12 intervention lessons. The outline was reviewed and revised with CSSC members, the CAB, local staff, and content experts (e.g., cultural consultant and physician with extensive knowledge of pediatric diabetes). Feedback was minimal and included specific suggestions for incorporating local knowledge. The Curriculum Team then created first lesson drafts by reviewing activities/content in programs described in Stage 2 for possible integration or adaption into TOD, and adding original content for topics that addressed community needs and assets. Content was also informed by findings in the scientific literature. The Curriculum Team reviewed draft lessons with the local study team. In some instances, the local team was asked to come up with additional ideas for lesson activities, which were incorporated into next drafts. These lessons were then reviewed by CAB and CSSC members; using their feedback, the Curriculum Team revised drafts and finalized/refined content in collaboration with local study team and/or CAB members and/or content experts (e.g., cultural consultant and diabetes/nutrition experts). It was determined that cultural elements that were not appropriate to be incorporated into the curriculum would be delivered orally by family health coaches during lessons. Penultimate drafts were reviewed and feedback collected from in-person CAB meetings, the CSSC, and local study staff prior to finalization.

The resulting TOD_SW program curriculum is summarized in Table 1. The program engages youth ages 10–19 with or at-risk for T2D and an adult “support person.” Lessons are taught by a Family Health Coach (FHC), a Native paraprofessional trained in delivery of TOD.

**Table 1:** TOD Southwest curriculum outline.

**Lesson**	**Topic area**	**Content**	**Examples of inclusion of indigenous knowledge and values**
1	Ready, Set, Go! Setting My Goals	FHC meets with Youth Participant, SP and other family members to review TOD Program structure and expectations. They also learn goal setting and set a long-term and short-term goal related to healthy living.	•Interconnectedness
2	Making My Healthy Plan	FHC works with youth to help them better understand the diabetes process in the body and address any of the youth's barriers in managing or preventing diabetes.	• Inclusion of cultural perspective of diabetes • Honesty • Bravery
		Optional: FHC, Youth, SP and Provider meet to discuss youth's diabetes plan.	
3	Eating to Win! A Balancing Act	This lesson introduces youth to energy balance. Youth learn about sources of energy for our bodies, understand how much energy our bodies need and, by the end of the session, are able to identify the amount of energy in many foods.	• Balance • Holistic teachings • Traditional foods
4	The SPIRIT Approach to Tackling Problems	Youth learn problem solving utilizing the SPIRIT approach (S-Stop and relax; P-understand the Problem; I-Identifying possible solutions; R-Review each solution; I-I choose this solution; T-Try it out and Treat yourself.). By the end of the session youth can identify and tackle problems, including barriers to achieving goals.	• Resilience • Interconnectedness
5	Think Positive!	Youth learn ways to think positive, turn a negative situation into a positive experience and understand the contribution a positive outlook has in living a healthy life. They also work with their FHC to create a positive sense of self.	• Positivity • Respect for self • Strength • Community values incorporated into personal power statements
6	Building a Winning Team	Youth work with FHCs to identify people in their support network who can help them overcome barriers to achieve goals, address problems, motivate them for success, and build self-esteem	• Interconnectedness • Community support • Importance of family
7	Sweet Revenge! Don't Be Fooled!	Youth work with their FHC to learn about carbohydrates, mainly sugar in their food and the difference between a lot of traditional foods and contemporary foods in their sugar content. They learn about hidden sugars and complete activities in which they can visualize sugar in foods.	• Traditional foods, importance of water • Food's connection to all living things • Food's connection to one's cultural, mental and emotional health
8	Let's Move: Exercise and Me	Youth learn the meaning of physical activity and different types of physical activity they can integrate into their daily routine. They learn what happens in our bodies during exercise and discuss the difference between sedentary activity and exercise. They learn about spiritual and cultural importance of running.	• Traditional forms of physical activity • Showing respect for our bodies • Balance • Connection of specific types of PA to cultural and spiritual wellness • Mindfulness
9	Finding My Groove	Youth work with the FHC to discover physical activities they like to do and understand how they can integrate these into their weekly schedule with support of family members	• Traditional forms of physical activity • Support from family • Gratitude
10	Eating to Win	Youth learn the importance of “My Plate,” portion size, food groups and meals. They learn about the function of different vitamins and minerals and will revisit the concept of energy in = energy out. They discuss where food comes from and how all things are connected to the earth.	• Natural foods, traditional foods
11–12	Yes I Can! Series	Take Control (Social Development and Diabetes)—Youth learn strategies to promote healthier choices when attending parties and going out to restaurants to eat. They discuss mindfulness when eating	• Mindful Eating • Appreciating the nutritional, cultural, and medicinal value of foods
		Say No (Dealing with Peer Pressure)—Youth learn about assertive, aggressive, and passive communication. They develop skills that help them say no to peer pressure and build the strength to stand up for what they believe when faced with a difficult situation.	• Bravery • Respect for others and self
		Plan for Success (Meal Planning)—Youth work with FHCs to come up with a strategy to meal plan. They also learn about the importance of being intentional when eating.	• Traditional foods and preparation • Eating seasonally
		Manage my Diabetes (Diabetes Management)—Youth with diabetes may complete this lesson. They learn about managing their diabetes and work with their family health coach to identify and overcome barriers to diabetes their management.	• Self-Care • Interconnectedness • Bravery • Honesty
**Four optional visits during first 6 months**			
	Social support visits	FHCs conduct a Social Support Visit with a participant if they arrive to a scheduled visit and it is apparent that the participant is not ready to discuss curriculum content due to difficult circumstances in their life. FHCs will be trained to provide support and refer the participant to appropriate resources in the community. Four Social Support visits are included in the curriculum structure. If additional support is needed, the case is discussed with the study team.	
**Monthly check-ins during second 6 months**			
16–22	Monthly check-in	FHCs schedule monthly check-ins with the participant and support person. These meetings are used to teach curriculum topics that were not covered during the 6 month core sessions, provide additional support to the participant and their families, and work to transition the support provided by the FHC during TOD to the support person.	

The program addressed multiple levels of influence (e.g., individual, family, community) using: (1) youth curriculum (details below); (2) support person curriculum (four lessons delivered during the first 6 months of the intervention and focusing on active listening, support skills, nutrition and physical activity), (3) engagement with local healthcare providers (e.g., transporting to clinical appointments, FHCs attending appointments with youth when possible, sharing reports with local providers as chart notes, presenting during pediatric provider meetings, etc.), (4) community connections and events (e.g., hosting monthly events like fun runs/walks, cooking lessons, dance classes), and (5) optional social support visits from FHCs. The youth curriculum included an intervention phase and a maintenance phase. The intervention phase included 12 lessons, each lasting about 45–60 min, delivered biweekly during the first 6-months of enrollment. All lessons were delivered in the home to the youth, and support persons were encouraged to attend. The 12 intervention lessons focused on balance and moderation and provided enrolled youth with knowledge and skills regarding nutrition, physical activity, diabetes, and life skills such as positive thinking, problem solving, support networks and communication. All lessons included the incorporation of Motivational Interviewing to help youth make and reach realistic healthy living goals based on information youth had learned, and a check in time to follow up on previous goals (43). Lesson activities included games, discussions, hands-on learning, and animated videos developed by local AI youth to reinforce lesson objectives through a serial drama. The first 10 intervention lessons were the same for all youth; for visits 11 and 12, the FHC worked with the youth to choose 2 of 4 lesson options based on their interests and progress through the program. These 12 intervention lessons were followed by a 6 month maintenance phase during which the FHC met with the participant monthly for about 20–30 min. These lessons included the youth, their support person, and the FHC and were designed to help youth sustain behaviors and transition support provided by the FHC through the TOD program to their support person.

Many of the community-driven targets within TOD curriculum are paralleled by existing or emerging scientific evidence. For example, TOD emphasizes balance and holistic wellness. This includes balancing energy in (food) with energy out (physical activity), overall wellness across physical, spiritual, emotional and mental health. Holistic health considerations are also critical to T2D outcomes. For example, those living with T2D are more likely than people without diabetes to experience depression, which in turn is associated with a variety of consequences such as increased rates of hospitalization and health care spending, activity impairment, comorbidities, and heightened mortality (44–47). Similarly, reports of apathy among Indigenous adult patients with T2D has been linked to lower quality of life and worse blood glucose management, while connection to cultural/spiritual ways is inversely associated with apathy (48).

As noted previously, the home-based, family-focused nature of TOD recognizes the centrality of family in many AI communities. Home-based delivery also addresses community-cited transportation issues that diminish access to care. For example, prior research shows that AIs report transportation barriers to healthcare twice as often as Whites (39% vs. 18%) and transportation was a contributor to intervention dropout in the AI DPP (49, 50). Our FHCs were pivotal in meeting participants “where they were at” by providing lessons where participants were most comfortable: in or outside their homes, at the TOD office, or at another communal location of their choosing.

Past research suggests that cultural and communication differences between AI patients and non-AI providers may compromise T2D care, and AI adults have reported feeling inadequately supported in T2D management (51–55). Loss of support has been viewed as a determinant of disease for some Indigenous people where disconnection leaves one “open to illness…from the outside world” (29). Community-based team members shared that their own experiences with prior T2D care was overly focused on individuals and medical aspects of disease and acknowledged benefits of a supportive AI FHC to deliver education in homes in culturally appropriate ways.

### TOD_SW Stage 4: Initial Program Evaluation Work

Implementation and evaluation of TOD included hiring and training FHCs to deliver the curriculum and conduct evaluation work. Training focused on curriculum delivery, study protocols, and special topics (often hosted by Native organizations), including motivational interviewing, facilitation skills, cultural teachings, diabetes knowledge, and Native nutrition. Follow-up trainings were conducted based on needs of FHCs identified through feedback on anonymous surveys and through regular check-in calls with all study team members.

The CSSC and Baltimore-based team members collaborated to identify TOD_SW evaluation methods. Because there were no other services or programs for youth with T2D and because TOD was a new program, we felt it was unethical and premature to conduct a randomized controlled trial to assess program efficacy. Thus, we designed a pilot pre/post evaluation study to assess program impact and acceptability.

Evaluation outcomes were selected by reviewing scientific literature and measures previously used in CAIH behavioral intervention studies, from which we created a matrix of potential measures (diet, A1c, zBMI, physical activity) to guide discussion at CAB and CSSC meetings. Community-based team members suggested inclusion of resiliency and medical home measures. We piloted measures with ~20 youth and their caregivers. Based on feedback, we made edits to the language of existing measures to improve face validity and comprehension (56, 57).

A total of *N* = 256 youth ages 10–19 years and *N* = 226 adult support persons enrolled in the study. Baseline data was collected from all youth enrollees. A total of 217 (84.8%) youth completed the 12-month evaluation. Youth completed an average of 9.35 lessons, with 73.4% of youth completing >8 lessons. A total of 21 FHCs across the four tribal community sites delivered 3,118 lessons and 1,149 evaluations during the project period. Sites also hosted regular community-based wellness activities (e.g., cooking demonstrations, walks/runs, biking). Over 30 outreach activities and 10 diabetes support services were conducted at community sites during the project period.

Throughout implementation, the Baltimore and Albuquerque teams met weekly with each site team to discuss case load, identify barriers to implementation and generally talk through what was and was not working in relation to the TOD program. The Baltimore and Albuquerque teams also led a cross-site teleconference meeting with all four sites once a month. This meeting was a time for sites to share successes and challenges. During these calls, we identified and made minor edits to the curriculum (e.g., creating a step-by-step visual of how diabetes happens in the body) and to the evaluation outcomes (e.g., collecting physiological measures from the Support Person) (58).

Findings provided promising evidence for the effectiveness and acceptability of TOD. Youth reported a significant increase in diabetes-related knowledge and pediatric quality of life from baseline to 12 months post intervention (59). The proportion of youth with depressive symptoms, those with hypertension and those reporting no days of physical activity in the past 3 days significantly decreased from baseline to 12 months post intervention. Youth experienced a significant decrease in zBMI, and youth with T2D at baseline experienced a significant decrease in A1c from baseline to 12 months post intervention. We also observed that a subset of adult support persons (*n* = 35) experienced a significant decline in BMI (58).

Reported program acceptability and overall satisfaction among support persons was high (58). Youth satisfaction was also high with 95.6% reporting they *learned a lot*, 95% reporting the program *helped them a great deal* and 96.13% reporting they would recommend this program to others. Acceptability was also high among youth with 87.2% reporting the number of visits with the FHCs was “just right,” 87.2% reporting the length of the program was “just right,” and 83.4% reporting visits with the health coach were “important” to their life.

The value and importance of FHCs who are members of the communities they serve cannot be overstated. Like many community health workers, FHCs shared similar lived experiences with participants and had invaluable cultural knowledge, both of which likely factored into overall satisfaction and retention. As one example, FHCs were able to incorporate traditional teachings that were not included in the curriculum. One FHC stated: “*Several families were already familiar with this teaching so it wasn't necessarily us sharing with them. Rather, it came up organically during certain lessons and FHCs related this discussion to TOD teachings*.” FHCs thus could tailor the program in culturally safe ways that acknowledge the diversity of Indigenous spiritual and cultural beliefs, which can vary dramatically even within specific communities. We also learned that in some instances, youth engaged in the program and worked toward behavior change because they considered the FHCs “cool;” FHC relatability to youth thus may influence program acceptability.

### TOD_SW Stage 5. Next Phase Efficacy Evaluation

The TOD_SW team has applied for funding to implement the program with expanded focus on mental health outcomes and a more rigorous evaluation design. In addition, a description of a “next iteration” efficacy trial currently underway in the Great Lakes region is described in detail below.

### TOD_SW Stage 6. Dissemination of Findings, Sharing, and Scaling

Findings from TOD_SW study were disseminated to participating communities through various methods. First, local study teams presented findings at a CAB meeting in their community. Second, letters were sent to participants presenting findings. Third, a report of findings along with all journal articles about the TOD program were reviewed and approved by local governing bodies. Fourth, the study team presented final TOD findings at the bi-annual Navajo Research Conference. Fifth, local Site Coordinators included TOD findings in their regular updates to local governing bodies. CAIH posted study findings on their website and disseminated findings to partners through their monthly newsletter. They also presented outcomes at scientific conferences including the biannual International Meeting on Indigenous Child Health and the annual meeting of the Society for Prevention Research.

### TOD_SW Stage 7: Ongoing Refinement, Next Generation Programming

We noted at the outset that the stage-based framework of intervention development and evaluation underpinning this work is dynamic and may not always follow a linear pattern. Beyond the processes described throughout the TOD_SW pre/post study, iterative changes and focal shifts continue. For instance, one of the TOD_SW community's Indian Health Service Units asked the CAIH team to translate the program to be implemented throughout school health clinics. The adapted TOD program, “Yéégo” (meaning “with great effort, do it” or “with spirit”), is currently being implemented. The TOD program has also been implemented in Native communities in California. This stage also interacts with Stage 5 (next generation efficacy trials) as we implement the TOD program in the Great Lakes region.

## Program Iteration #2: TOD Great Lakes (TOD_GL)

The foundational work of the TOD_SW team resulted in a promising multigenerational home-based diabetes preventive intervention developed by and for Indigenous Peoples. Below, we describe a next-generation iteration of TOD development and evaluation using Figure 1 as a guiding framework.

### TOD_GL Stage 1: Invitation and Description of the Team

In June 2019, a team of Ojibwe community and JHU-based researchers working in the midwestern US received funding to adapt, implement, and evaluate the TOD program through a wait-list RCT (TOD_GL). The team had already completed two observational studies focused on Ojibwe experiences with a) mental health and diabetes (Mino Giizhigad study), and b) stress and diabetes (Gathering for Health study); additional background information for these studies has been published previously (60, 61). In alignment with CBPR approaches a longstanding goal of the team was to translate their research findings into action in the form of public health programming (62). After reviewing existing literature on diabetes preventive interventions, the team invited TOD_SW researchers to a local meeting to present information about the program and share preliminary outcome data. Additional information on the formative work surrounding TOD_GL is noted in Stages 2 and 3 below.

The TOD_GL team includes JHU researchers at the CAIH Great Lakes Hub offices in Minnesota and Community Research Councils (CRCs) from five participating Ojibwe reservation communities. Each CRC is comprised of four to eight individuals who act as equal partners with academic researchers in every stage of program adaption, implementation, and evaluation (59). The term *Community Research Council* was adopted to replace *Advisory Boards* to acknowledge the substantial and active contributions of the CRCs in the project that goes far beyond advising. CRCs include representatives from participating communities who have diabetes or have family members with diabetes, work at local clinics, and/or are Elders, nurses, diabetes educators, fitness coaches, and representatives of tribal governments.

### TOD_GL Stage 2: Basic Research and Formative Work

The GL team's prior research on mental health, stress, and T2D generated foundational basic/observational knowledge that informed TOD adaptation processes. In particular, our team generated scientific evidence that aligned with stress process models of disease (63, 64) to suggest: a) stressors, including exposure to discrimination, trauma, family stress, and health-related strains, are associated with worse health and diabetes-related outcomes among Ojibwe adults living with T2DM; and b) coping factors like family and community support and Ojibwe cultural factors (e.g., identity, communal orientation, etc.) offset or buffered the negative impacts of stress on health and/or served as protective factors for diabetes patients (48, 65, 66). Given this, one component of TOD_GL program adaptation focused on inclusion of stress/coping curricular content.

Additional formative work for the GL team included CRC-led community feasts and forums, listening sessions, brief surveys, and group discussions at all 5 tribal sites prior to applying for funding for the TOD_GL trial. These activities generated preliminary community-based data to suggest that residents across the five tribes were interested in the content of the program, found it matched gaps in terms of their needs/their community needs, and that they gave high rankings of perceived effectiveness and willingness to participate in the program.

### TOD_GL Stage 3: Program Adaptation and Refinement

The TOD_GL curriculum is rooted in the lessons, structure, and foundational successes of TOD_SW. The process of adapting curriculum was iterative and centered Ojibwe community and cultural perspectives in alignment with Okomoto et al.'s (33) description of deep-structure cultural adaption. We used findings from our observational studies to create two new lessons to address historical trauma, healing, stress, coping, and the relationship of these factors to diabetes. Additional changes were made to the curriculum to enhance the presence of Ojibwe-specific cultural knowledge and strengthen existing content related to values such as connection to the earth, the importance of family and community, and drawing on the strengths of past generations to forge a better future. We also shifted the primary “target” of intervention to adults living with T2D who would enroll in the program alongside a youth who lived in their home; that is, adults with diabetes would enroll in the program for intervention purposes, and youth would enroll with a primary focus on preventing diabetes. Based on community input, a foundational premise of TOD_GL was that adult-child interaction is a leverage point for family-based motivation (67). This shift required basic changes to curriculum examples, language, and removal of “support person” content that had been used in TOD_SW. In addition, CRC members recommended renaming to call the program *Together Overcoming Diabetes* in English. The resulting TOD_GL intervention includes 14 core lessons, six maintenance lessons, and four optional social support visits. Following TOD_SW, the intervention is delivered to adult-youth dyads in their homes by local FHCs. Table 2 summarizes the TOD_GL curriculum.

**Table 2:** TOD Great Lakes curriculum outline.

**Lesson**	**Title**	**Content**	**Examples of inclusion of indigenous knowledge and values**
1	Diabetes 101 and Goal Setting	FHC and adult-youth dyad meet to learn about risk factors for T2D, how diabetes occurs in the body, and how to use goal setting as a tool for preventing or management diabetes.	• Bravery as an Ojibwe cultural teaching to promote T2D management
2	More Information on Diabetes	FHC and adult-youth dyad meet to review how diabetes occurs in the body and learn about the long-term effects of diabetes complications, how diabetes can be controlled, A1c, how to check blood sugar, and role of diabetes medications.	• Revisiting bravery and honest communication with self, others around T2D care
3	Historical Trauma and Healing	Focus is on the impact of historical trauma on Indigenous communities and health. Explores connection between historical trauma and diabetes. Describes the role of traditional healing activities in helping people heal and guides families through traditional healing practice (if family is interested).	• Use of traditional practices for healing • Elder's teachings on traditional healing practices
4	Stress and Diabetes	Understanding relationships between stress and diabetes. Topics covered include the difference between stress and stressors, strategies for relieving stress, the benefits of meditation, a guided visualization activity, and an opportunity for the participants to reflect on their support network.	• Cultural connection as a protective factor • Elder's teaching on dealing with stress • Drawing on community support
5	Nutrition 101	This lesson is the first of five lessons that focuses on nutrition. In this lesson, adult-youth dyads are guided by an FHC through content about different food groups, the body's energy balance, how to build a balanced plate of food, and how to read nutrition facts labels.	• Elder's teaching on traditional foods • Examples of traditional foods for each food group • Importance of traditional food for spiritual, nutritional, and cultural health • Connectedness to all living things
6	Exercise Effects and Safety	This lesson is the first of two lessons that focuses on physical activity. In this lesson, FHCs and adult-youth dyads learn about sedentary v. active behaviors, what happens to our bodies when we exercise, and how to stay safe while exercising.	• Traditional forms of physical activity • Showing respect for our bodies
7	The SPIRIT Approach to Problem Solving	FHC and adult-youth dyad meet to learn about strategies for overcoming challenges. This lesson uses the SPIRIT problem solving framework and offers the participants a chance to practice using this approach for a problem they are facing in their own lives, or in the lives of fictional characters.	• Reinforcing culturally-grounded relaxation techniques
8	Mindful Eating in cultural context	In this lesson, participants are guided by their FHC through content and activities related to savoring foods, the difference between mindful and mindless eating, understanding portion sizes, recognizing when you are full, and food journaling.	• Mindful eating with traditional foods • Appreciating the nutritional, cultural, and medicinal value of foods • Encouragement to food journal using Ojibwemowin
9	Building Positivity	FHC and adult-youth dyad meet to learn about how appreciating the good things in our lives, and how to strengthen positive thinking in their lives.	• Respect for self • “Appreciating our strengths” in context of fulfilling our family and community responsibilities • Shift from “self-esteem” to consider cultural emphasis on humility • Elder's teaching on taking care of self
10	Not All Foods are Created Equal	FHCs and adult-youth dyads learn about the six major nutrients, vitamins and minerals, empty calories, whole grains, sugar sweetened beverages, and the importance of water.	• Eating minimally processed foods (like traditional foods) • Importance of water
11	Let's Get Moving	In this second lesson about physical activity, participants and FHCs learn content related to overcoming common obstacle to exercise including how to exercise without a gym, drawing on the support of others to help in your exercise journey, finding time to fit in exercise, and exercising as a family.	• Respect for our bodies • Traditional forms of exercise • Drawing on the support of family and community
12	Communication	The FHC and adult-youth dyad meet to learn about non-verbal communication, body language, different communication styles, and active listening.	• Elder's teaching on talking circles
			• Protecting one's community by being a warrior against violence and bullying
13	Putting Nutrition Knowledge Into Practice	Participants and FHC explore content around cooking, meal planning, strategies for eating healthy on a budget, and eating seasonally.	• Traditional food preparation methods • Eating seasonally
14	Focus on Family in Community	The adult-youth dyad and FHC learn about the importance of eating with others, eating well as a family, and strategies for eating well when at restaurants or at community or family events.	• Drawing on and supporting, family and community • Love
**All lessons include Ojibwemowin vocabulary lessons**			
Four additional optional visits during first 6 months			
	Social support visits	FHCs conduct a Social Support Visit with a participant if they arrive to a scheduled visit and it is apparent that the participant is not ready to discuss curriculum content due to difficult circumstances in their life. FHCs trained to provide support and refer the participant to appropriate resources in the community. Four Social Support visits are included in the curriculum structure. If additional support is needed, the case is discussed with the project team.	
Maintenance visits			
16–22	Monthly check-in	FHCs schedule monthly check-ins with the participant and support person. These meetings are used to review curriculum topics that were covered during the first 6 months of the program, provide additional support to the participant and their families, and work to build skill among the adult-youth dyad to support each other on their healthy living journeys.	

The process of adapting and refining TOD_GL curriculum involved deep reviews and demonstrations of draft lessons by/with community/university teams, advisement by elders, and reviews and consultations with outside experts and medical clinic staff in each community. This process varied based on the objectives of specific lessons. For example, lessons 1 and 2 focus on understanding the biomedical mechanisms of diabetes and its management and thus were shared with local clinic staff early in our adaptation process. Staff reviewers included diabetes educators, nurses, administrators, physicians, and fitness coaches who gave us feedback on the medical accuracy and alignment of lessons with local clinical protocols. As another example, five nutrition lessons were reviewed by CRCs during a full day in-person meeting. Team members reviewed each lesson in small groups and provided feedback on necessary cultural adaptions and general improvements. Refinements included further integration of traditional Ojibwe foods and processing techniques, inclusion of the role and importance of traditional foods in Ojibwe spiritual and social traditions, and strengthening the focus on the interconnectedness of people with nature (i.e., recognition of the plants and animals that feed and nourish us).

A respected Ojibwe Elder Lee Obizaan Staples provided Ojibwemowin (Ojibwe language) words and phrases relevant to each lesson and cultural teachings on topics such as traditional cultural practices, dealing with stress, and taking care of oneself. The critical need for Ojibwemowin throughout the curriculum has been reinforced by FHCs, who set aside weekly meeting time for language lessons and practice time. The TOD_GL team also included and cited a variety of regionally relevant content from published and online materials from Indigenous-serving organizations, activists, chefs, and authors. All curricular revisions were facilitated by a project coordinator and drafted in collaboration with the JHU Curriculum Team, most of whom had worked on TOD_SW. University and community members reviewed and provided input on all lessons after adaptations and revisions were completed prior to pilot sessions.

### TOD_GL Stage 4: Initial Program Evaluation Work

Our focus at Stage 4 was to ensure basic acceptability and comprehension of the revised TOD curriculum. All lessons were piloted with Indigenous families from each of the participating communities (*N* = 15 participants). Pilot families included one adult and at least one youth aged 10–16 years. All pilots were conducted in late 2020 and facilitated virtually to align with COVID-19 safety protocols put in place by JHU and tribal agencies. Pilots lasted between 30 and 90 min and involved a JHU team member completing a verbal consent process before guiding participants through 1–2 individual lessons. After completion of the lesson, JHU staff asked families a series of questions soliciting feedback on sections that were confusing, hard to follow, whether or not cultural teachings or language flowed or was appropriate to the lesson, if anything important was missing. Families received a $50 gift card for participating in pilot sessions.

### TOD_GL Stage 5: Outcome Evaluation

As of this writing, the TOD_GL team has officially launched a wait-list randomized control trial (RCT) with a randomized block design by reservation community to evaluate program effectiveness. We chose a wait-list design (as opposed to a classic RCT where the control group foregoes treatment) in alignment with cultural and community goals of sharing, inclusion, and equitable distribution of benefits. Primary and secondary outcomes for the trial are HbA1c and lipids (adult participants); and BMI/zBMI, depressive symptoms, physical activity, nutrition, and coping resources and responses (e.g., family support, engagement with cultural activities, stress management) (adult and youth participants). We anticipate families will continue enrolling in the trial through summer, 2022, and we will gather evaluation data at baseline, 3, 6, 12, 18, and 24-month intervals. In addition, we plan to complete a participatory evaluation activity called ripple effects mapping with families and community members across sites twice throughout the duration of the study.

### TOD_GL Stage 6: Dissemination of Findings: Sharing and Scaling

A mission of the CAIH and a goal of our teams is to partner with Indigenous communities to improve health and well-being. As such, we are strategic about prioritizing dissemination of eventual findings and lessons learned along the way using techniques that move beyond conventional academic publications. In addition to empirical manuscripts, our team produces and presents Technical Reports to partnering Tribal Governing Boards/Councils and reservation-based health and human service workers and grant writers. We create public webpages, use social media, and have a project YouTube channel to share information about the project and eventual results. If TOD is found effective through this research, our CBPR design will expedite program uptake and integration with existing services within communities and our attention to dissemination possibilities will promote sharing with other Tribal and Urban Indian groups and organizations.

### TOD_GL Stage 7: Ongoing Refinement, Next Generation Programming

The TOD_GL team is following the lead of the SW experience: curriculum refinement continues as FHCs provide feedback on lessons “live,” as they are learned and rolled out within their own communities. For example, Lesson 13 is being adapted to provide more content for those who get their groceries via food assistance programs. FHCs recognized that the current version of the lesson was geared toward those who were able to shop for their groceries and therefore have control over what foods are available in their homes. In addition, the curricular adaptations made for TOD_GL have been shared back to SW teams, who in turn are working to implement these changes within their own communities.

## Discussion

The TOD program has been developed, evaluated, and iteratively refined across multiple Tribal communities over the span of a decade. While we have taken distinctive paths through the stages of intervention development and evaluation displayed in Figure 1, the two iterations of TOD described in this manuscript share important commonalities that we view as key markers of successful collaboration. These include investing time and resources in community-university partnership building and the creation of systems and structures to support shared work (e.g., CRCs, CABs, CSSCs, etc.), community-driven identification of priorities for programs and outcomes, attention to and incorporation of general and local Indigenous knowledge, and employment of FHCs who are members of the communities with which they work (60). All of this work requires attention to what has been called a “flexibility imperative” (23), both in terms of agility to address unique needs across communities, and being prepared to facilitate necessary shifts in curriculum content, methodological processes, and target outcomes as a study unfolds.

The need for such flexibility is of course made more challenging in the context of sponsor/funder, institutional, and local pressures to address urgent issues within what can sometimes be stringent milestone metrics and budget constraints. As others have argued, there is need for expansion of markers of “success” in health equity research to include partnership synergy, infrastructure development, resource sharing, and sustainability efforts via flexible timelines and funding structures (23, 68). Further, challenges related to the COVID-19 pandemic continue to impact the TOD_GL trial in several ways: a delayed launch, missed and delayed study milestones, and difficulties securing adequate staffing all exist vis-à-vis significant losses and stressors related to the pandemic for all study staff and TOD families and the ongoing, heavy toll of the pandemic on Tribal and public health infrastructure (69).

There are also distinctions between the two iterations of TOD worthy of attention. While Stage 7 is important for flexibility and applicability of the program over time and across groups, this can also make comparability across distinct iterations of a curriculum difficult. For instance, we do not know if the addition of content around historical trauma, stress, and coping in TOD_GL will activate unique mechanisms for health promotion or change compared to the SW iteration. In addition, the complexities navigating five distinct clinical systems, dozens of diabetes healthcare providers, and practical constraints of COVID-19 mean that TOD_GL FHCs are not as connected to local healthcare providers or systems as was done in the SW. Despite these differences, both iterations of the program share a similar structure and target common “active ingredients” for health improvement (e.g., family-based changes, connection to culture and support systems, goal-setting, knowledge sharing, addressing barriers to care, etc.). Furthermore, our stage-based process of program development and implementation is potentially useful across community contexts, including in non-Indigenous communities.

A number of questions and areas for future exploration remain. We found that 62% of TOD_SW lessons were attended by at least one family member in the household who was not officially enrolled in the study. This finding suggests to us that inclusion beyond target dyads may allow for more deliberate family systems changes. Additional studies are needed to better understand the role of the family and extended family engagement on the efficacy of TOD. Sustainability of the program within Tribal or urban Indian or non-Native community settings beyond grant funding periods is not fully determined (billing for FHCs, program costs, etc.). Perhaps most importantly, major efficacy questions remain to be answered with regards to the impact of TOD on diabetes outcomes for Native families. Some of these questions can be empirically answered in planned and ongoing trials.

We also recognize the need for humility in our work. TOD as a single program does little to address structural or environmental determinants of health, many of which are rooted in historically anchored traumas and policies that translate into tangible issues in modern times (16). As one FHC commented, “youth were not exposed to many healthy foods, specifically vegetables, because they weren't available in local stores.” While behavioral interventions hold promise for improving health at individual and family levels, systematic efforts to address ongoing marginalization and promote equitable access and policies surrounding healthy food systems and culturally safe services and healthcare are required for lasting impacts on health equity.

## Data Availability Statement

The original contributions presented in the study are included in the article/supplementary material, further inquiries can be directed to the corresponding author.

## Author Contributions

MW created an initial model and outline for this manuscript and led a collaborative writing process. RC drafted significant sections of the manuscript and helped with final edits. All remaining authors contributed equally to the work by assisting with literature reviews, writing sections of the manuscript, and providing comments on final drafts.

## Funding

Research reported in this article was supported by the National Institute of Diabetes and Digestive and Kidney Diseases of the National Institutes of Health (DK091250), the Shakopee Mdewakanton Sioux Community, and by the Bristol Meyers Squibb Foundation.

## Author Disclaimer

The content is solely the responsibility of the authors and does not necessarily represent the official views of the National Institutes of Health.

## Conflict of Interest

The authors declare that the research was conducted in the absence of any commercial or financial relationships that could be construed as a potential conflict of interest.

## Publisher's Note

All claims expressed in this article are solely those of the authors and do not necessarily represent those of their affiliated organizations, or those of the publisher, the editors and the reviewers. Any product that may be evaluated in this article, or claim that may be made by its manufacturer, is not guaranteed or endorsed by the publisher.
